# Contrasting cumulative risk and multiple individual risk models of the relationship between Adverse Childhood Experiences (ACEs) and adult health outcomes

**DOI:** 10.1186/s12874-020-01120-w

**Published:** 2020-09-29

**Authors:** Marianna D. LaNoue, Brandon J. George, Deborah L. Helitzer, Scott W. Keith

**Affiliations:** 1 College of Population Health, Thomas Jefferson University, 901 Walnut St., 10th Floor, Philadelphia, PA 19107 USA; 2 College of Health Solutions, Arizona State University, 500 N 3rd St, Phoenix, AZ 85004 USA; 3 Division of Biostatistics, Department of Pharmacology & Experimental Therapeutics, Thomas Jefferson University, 1015 Chestnut Street, Suite 520, Philadelphia, PA 19107 USA

**Keywords:** Adverse childhood experiences, ACEs, Childhood adversity, Model selection, Model comparison, Behavioral risk factor surveillance system, BRFSS

## Abstract

**Background:**

A very large body of research documents relationships between self-reported Adverse Childhood Experiences (srACEs) and adult health outcomes. Despite multiple assessment tools that use the same or similar questions, there is a great deal of inconsistency in the operationalization of self-reported childhood adversity for use as a predictor variable. Alternative conceptual models are rarely used and very limited evidence directly contrasts conceptual models to each other. Also, while a cumulative numeric ‘ACE Score’ is normative, there are differences in the way it is calculated and used in statistical models. We investigated differences in model fit and performance between the cumulative ACE Score and a ‘multiple individual risk’ (MIR) model that enters individual ACE events together into prediction models. We also investigated differences that arise from the use of different strategies for coding and calculating the ACE Score.

**Methods:**

We merged the 2011–2012 BRFSS data (*N* = 56,640) and analyzed 3 outcomes. We compared descriptive model fit metrics and used Vuong’s test for model selection to arrive at best fit models using the cumulative ACE Score (as both a continuous or categorical variable) and the MIR model, and then statistically compared the best fit models to each other.

**Results:**

The multiple individual risk model was a better fit than the categorical ACE Score for the ‘lifetime history of depression’ outcome. For the outcomes of obesity and cardiac disease, the cumulative risk and multiple individual risks models were of comparable fit, but yield different and complementary inferences.

**Conclusions:**

Additional information-rich inferences about ACE-health relationships can be obtained from including a multiple individual risk modeling strategy. Results suggest that investigators working with large srACEs data sources could empirically derive the number of items, as well as the exposure coding strategy, that are a best fit for the outcome under study. A multiple individual risk model could also be considered in addition to the cumulative risk model, potentially in place of estimation of unadjusted ACE-outcome relationships.

## Background

A very large body of research documents relationships between Adverse Childhood Experiences (ACEs) and adult health outcomes. Much of the data in which these inferences are based comes from cross-sectional surveys containing adults’ retrospective self-reports of their ACEs and concurrent reports of their health status. We refer to this type of design and data structure as the ‘ACEs Framework’ [1] and to questionnaire responses over a specific set of adversity events contained in these datasets as srACEs (self-reported ACEs). While this tradition arguably began with the landmark 1998 Felitti et al. Kaiser ACE’s Study [2], versions of the Kaiser group srACE questions are now used in several other large-scale health surveys including the CDC’s Behavioral Risk Factor Surveillance System (BRFSS) survey [3].

Remarkably, these studies show a substantial degree of inconsistency in the operationalization of the srACEs as a predictor variable. While a ‘cumulative risk’ conceptual model guides most research, resulting in the operationalization of childhood adversity using the cumulative numeric ‘ACE Score’, there are differences in the way this cumulative score is calculated and used in statistical models. Additionally, alternative conceptual models to the cumulative risk model, which can yield different conclusions about the effects of adversity on outcomes, are rarely used and even more rarely directly contrasted to each other. Differences in operationalization may impede efforts to synthesize the literature and differences in conceptual models of how adversity impacts outcomes have high stakes as policy and intervention programming depend on this body of literature. There has been some recent criticism of the use of the ‘ACE Score’ [4–6], some of it from within the original Kaiser ACEs Study team [7]. Such criticism tends to focus on using a ‘crude’ or oversimplified measure in policy-making. However the recent critical publications are conceptual reviews, not empirical reports. Large data sources such as the BRFSS survey represent a significant investment of research resources; the BRFSS effort specifically surveys over 450,000 individuals each year, with a yearly budget over $18 million [3]. These observations were the rationale for the present study.

In this paper we analyze two conceptual models of the effects of ACEs on adult health, contrasting the cumulative risk model (using a cumulative ACE Score) with a ‘multiple individual risk’ model that allows for each ACE event to have its own relationship with the outcome in a multivariable model that includes all the ACE event predictors. In order to undertake this comparison, we also analyze differences arising from the practical decisions that have to be made about which specific ACE questions to include from among those available and how to code individuals as ‘exposed’ when response options include information about the frequency of events.

### Models of the effects of adversity - Cumulative & Multiple Risks

#### Cumulative Risk - CR

In the ACEs framework literature, the dominant model of the effects of developmental adversity on later health is the cumulative risk model. This model holds that it is not so much specific events which are detrimental to health, but rather that it is an accumulation of events (regardless of which specific events they are) that confers risk for negative health effects [8]. The cumulative risk model is a specific type ‘multiple risk’ model [8] where exposure to multiple risks are included in the same statistical model. In the ACEs literature this is widely implemented through the use of a cumulative numeric score (the ‘ACE Score’) that indicates the total number of exposures. This model answers the question ‘*what is the impact of increasing numbers of events? (regardless of which events they were?’).* There are multiple ways to implement this model, however. Here we contrast a continuous with a categorical variable approach.

##### Continuous cumulative risk

A cumulative ACE Score can be treated as an integer count variable (i.e. a continuous variable) in statistical models. This model answers the question ‘*what is the impact of increasing numbers of events?’ (regardless of which events they were?)* but with a restrictive assumption about linearity of the effect (that each additional ACE has an equal impact). An example of the logistic regression model is represented in eq. (1) for the *i*^th^ participant, assuming a binary outcome and continuous ACE Score (1–11), and with the same set of covariates (not represented in the model equation).

1 $$ \ln \left(\frac{p\left( outcom{\mathrm{e}}_i\right)}{1-p\left({outcome}_i\right)}\right)={\beta}_0+{\beta}_1\left( ACE\ {Score}_i\right) $$

As an example in the BRFSS literature, Nurius, Logan-Green and Green used a total ACE Score (0–8) and reported significant coefficients of −.19 (healthy days) and .23 (mental health symptoms) [9], implying a constant decrease of .19 healthy days and constant increase of .23 mental health symptoms for each additional ACE reported. An ACE Score characterized in this way serves as the primary illustration of a dose-effect relationship: one in which the dose-effect relationship is constant across levels of the ACE score.

##### Categorical cumulative risk

The most common alternative characterization for the ACE Score is to use it as a categorical variable in prediction models. While the model answers the same question about *‘the effects associated with increasing numbers of events’,* it does not assume linearity of the relationship and allows each specific ACE count to have its own relationship with the outcome. The counts are entered into prediction equations as categories, producing a separate coefficient for each, compared to a reference category (usually zero). An example of the logistic regression model is represented in eq. (2) for the *i*^th^ participant, assuming a binary outcome and categorized and reference cell coded ACE Score (1–11).

2 $$ \ln \left(\frac{p\left({outcome}_i\right)}{1-p\left({outcome}_i\right)}\right)={\beta}_0+{\beta}_1\left(1\ {ACE}_i\right)+{\beta}_2\left(2\ {ACE s}_i\right)+\bullet \bullet \bullet +{\beta}_{11}\left(11\ {ACE s}_i\right) $$

The most common approach is categories of 0, 1, 2, 3, and ≥ 4, [10, 11] although a top category of ≥5 is also common [12, 13]. Other researchers have combined counts into other categories such as 0, 1–3, 4–6, 7–9 [14], or used a wider range of the variable (1–8 compared to zero) as a categorical predictor [15, 16]. Compared to a continuous ACE Score approach, this model is more flexible and yields a different inference about the dose-response relationship: that risk for the outcome increases monotonically, but not at the same rate for every additional ACE. This was the approach used in the first ACEs study publication [2], where a total of 17 individual questions were aggregated into 7 categories of events and a truncated categorical variable (0, 1, 2, 3, ≥ 4) was used in statistical modeling. Those results showed increasing odds across the levels of the categorical variable (compared to those in a zero ACEs reference category) of 1.1–2.2 for current smoking; 1.5–4.6 for two or more weeks of depressed mood in the previous year; and 1.1–1.6 for ‘severe obesity’ [2]. Results arrived at using this treatment of the ACE predictor are not only exceedingly common in the literature, but have been translated into public policy [17] as well as public-facing internet sources that refer to the risks associated with ‘4 or more ACEs’ [18, 19].

#### Multiple Individual Risks - MIR

In contrast to the cumulative risk model, the presence or absence of multiple separate ACE events can be included together as separate predictors in a single regression; we refer to this as a ‘multiple individual risk’ model. This model is not the same as analyzing univariate (unadjusted) associations between single ACEs and outcomes, which is a common feature in studies that use a cumulative risk model (ACE score) in their primary analysis. Instead, this model answers the question *‘what is the impact of the occurrence of each specific event (given the presence/absence of the other events)’?.* The model yields coefficients for each event separately, controlling for the other events in the model. It is therefore expected to function well for events which are highly correlated, as has been extensively supported for ACEs [20]. An example of the logistic regression model is represented in eq. (3), assuming a binary outcome and entry of all 11 BRFSS ACE events in the model.

3 $$ \ln \left(\frac{p\left({outcome}_i\right)}{1-p\left({outcome}_i\right)}\right)={\beta}_0+{\beta}_1\left( Household\ {Mental\ Illness}_i\right)+{\beta}_2\left({Household\ Alcoholism}_i\right)+{\beta}_3\left( Household\ {Drug\ Abuse}_i\right)+{\beta}_4\left({Household\ Criminal}_i\right)+{\beta}_5\left({Divorce}_i\right)+{\beta}_6\left({Household\ Violence}_i\right)+{\beta}_7\left({Physical\ Abuse}_i\right)+{\beta}_8\left({Emotional\ Abuse}_i\right)+{\beta}_9\left({ Sex ual ly\ Touched}_i\right)+{\beta}_{10}\left({ Sex ual\ Touching}_i\right)+{\beta}_{11}\left( Forced\ {Sex}_i\right)\kern7.8em $$

Despite the high degree of information contained in this type of model, it appears only rarely in the ACEs framework literature. Our review found only one instance in the BRFSS data, in a study examining individual and cumulative effects of ACEs on adult mental health. In that study, only the specific ACEs that had a significant univariate relationship with the outcome were included in the ‘multivariate’ models, and they found that different sets of ACE events had significant associations with the mental health outcomes under study [9].

We presume that the absence of this type of model in the literature is due to the fact that, while the model itself is additive with respect to the joint effects of the events on the outcome, this model does not contain general summary information about cumulative effects. That is, when effect estimates for specific individual ACEs are estimated in the same model, effect estimates for a specific *number* of events are not estimated and therefore the model does not produce specific information about a dose-response relationship. However, there are instances that it makes theoretical sense (either for certain types of adversity or for certain outcomes) to consider that an accumulation of adversity might not be the only model to consider. The review of Lacey and Minnnis provides an overview [6].

### Model comparisons

Choosing a predictor characterization directly impacts interpretations about the effects of adversity on outcomes, and the cumulative risk model and the multiple individual risk model yield different inferences. Only in the first case would we be able to infer that an accumulation of adverse events (regardless of which specific events) is related to outcomes in a dose-response manner. However, only in the second case are we able to infer that one or some specific ACE events are a strong predictor, compared to other ACE event types.

Similarly, treating the ACE score as continuous vs categorical in the CR framework also has implications for interpretation. Finding that each additional ACE event contributes in a constant linear way to risk for negative outcomes (e.g. [9, 21]) is a very different conclusion than finding that change in risk for an outcome is smallest across intervals at the low end of ACE Scores, but that increases in risk for the outcome are accelerated across intervals at the higher end of the score range (e.g. [22]).

However, even though these modeling choices can result in different conclusions, there is only limited evidence that directly contrasts them. Some exceptions include comparison of a latent class predictor characterization (LCA) to the cumulative risk ACE Score in predicting outcomes in college students [23] which found that LCA performed similarly to the cumulative ACE Score. In contrast, Schilling et al. found that a cumulative risk approach produced different predictions than treating the same data with a cluster analysis approach [24].

The cumulative risk model is a straightforward and easy to understand explanatory model that has helped to publicize the negative health effects of adversity, but it has both statistical and theoretical shortcomings [5, 25]. In this study, we investigated differences in model fit and performance based on operationalization of an ACE predictor variable in a cumulative risk model (with ACE score as continuous or categorical) vs a multiple individual risk model when applied to three commonly studied health outcomes.

## Method

### Data

We merged data from the 2011 and 2012 publicly-available Behavioral Risk Factor Surveillance System (BRFSS) cross-sectional, random-digit-dial telephone surveys conducted by health departments in all 50 US states in collaboration with the Centers for Disease Control [26]. Respondents are English and Spanish speaking adults aged 18 years or older, who are non-institutionalized, and live in a household with a working landline telephone or included cell phone. Only the states that administered the ACEs module in each year were included (16 states total).

### Self-report ACEs

The BRFSS survey contains 11 srACEs, prefaced with “Before the age of 18” …:

1. Did you live with anyone who was depressed, mentally ill, or suicidal? [*Household Mental Illness*]
2. Did you live with anyone who was a problem drinker or alcoholic? *[Household Alcoholism]*
3. Did you live with anyone who used illegal street drugs or who abused prescription medications? *[Household Drug Abuse]*
4. Did you live with anyone who served time or was sentenced to serve time in a prison, jail, or other correctional facility? *[Household Criminal]*
5. Were your parents separated or divorced? [*Divorce*]
6. How often did your parents or adults in your home ever slap, hit, kick, punch or beat each other up? *[Household Violence]*
7. Before age 18, how often did a parent or adult in your home ever hit, beat, kick, or physically hurt you in any way? Do not include spanking. *[Physical Abuse]*
8. How often did a parent or adult in your home ever swear at you, insult you, or put you down? *[Emotional Abuse]*
9. How often did anyone at least 5 years older than you or an adult, ever touch you sexually? *[Sexually Touched]*
10. How often did anyone at least 5 years older than you or an adult, try to make you touch them sexually? *[Sexual Touching]*
11. How often did anyone at least 5 years older than you or an adult, force you to have sex? *[Forced Sex]*

For items 1–5, response options are ‘yes’ and ‘no’. For questions 6–11 response options are ‘never’, ‘once’, and ‘more than once’.

### Outcomes

We selected three dichotomous outcomes: lifetime history of depression: *(Ever told) you that you have a depressive disorder, including depression, major depression, dysthymia, or minor depression?,* obesity status (BMI ≥ 30, calculated in the data from self-reported height and weight) and presence of cardiac disease (coded in the data by any affirmative response to ‘having had a heart attack’ or ‘having had angina’). Although not systematic, we choose these outcomes as they represent both mental and physical health states, and one, while still self-report (BMI) is calculated in the data.

We included the same covariates in every model, chosen as they represent common modeling decisions in the published BRFSS literature. For sex, age (5 categories), education (4 categories), income group (5 categories) and insurance status (has insurance vs not) we used the computed BRFSS variables (available in the BRFSS codebooks). For race (Black, White, and other) and marital status (married/member of an unmarried couple, divorced/widowed, and never married) we created new variables, collapsing the available BRFSS categories to address low response-frequency categories.

### Data Screening & Analysis

We included 56,640 cases with no missing data on any covariates or outcomes, and with no more than 1 missing ACE (82.1% of cases in the merged 2011–2012 data). Cases missing one ACE were imputed as ‘no’ (< 1% of cases). This analytic decision was made in order to ensure that model comparisons were made between models fitted in the same data. Data were screened to ensure that at least 20 cases were present in the cross-tabs of the ACE scores and the outcomes as well as the covariates.

Because of the substantial reduction in the total number of cases, we omitted the survey design variables from our modeling, as survey weights are calculated based in the full dataset. The dichotomous outcomes were modeled with logistic regression. Data were analyzed in R using the R Studio® IDE, [25] and the package ‘nonnest2’ [27] for model comparisons.

### Within-category models and model comparisons

The model comparisons of interest in this study are between the cumulative risk model (with the ACE Score used as either a continuous variable (CrCn) or a categorical variable (CrCat)), and the multiple individual risk (MIR) model. In order to make fair comparisons between those models, we first arrived at the best fitting model within each category. As noted, for 5 of the BRFSS ACE questions, response options are ‘yes’, and ‘no, while for the other 6 questions the response options are ‘never’, ‘once’, and ‘more than once’. Although it is possible to code ACE predictors that incorporate the frequency information, it is uncommon in the literature. Instead, investigators routinely define a cut-off to determine an exposure. In most cases responses of ‘once’ are sufficient, but in some cases ‘more than once’ is used [28]. In many published studies in the BRFSS data this decision is not noted [10, 14]. Additionally, even though there are 3 separate questions asking about some form of sexual adversity, in the majority of published ACEs research, an affirmative response to any of the 3 questions is used as a binary indicator of ‘sexual abuse’.

To arrive at the best-fit model within each category, we created different versions of the ACE predictor based on the permutations possible for exposure coding (‘once’, vs ‘more than once’) and number of items (9 questions vs 11) and iteratively arrived at the best-fit model for each outcome separately, through within-category pair-wise comparisons.

Within the CRCn models we allowed for non-linearity by estimating a model that included a quadratic term. Within the ‘multiple individual’ risk models, variance inflation factors were obtained for all ACE predictors to assess multi-collinearity.

### Between-category models and model comparisons

After the best-fit model was obtained for the MIR, CRCn, and CRCat model categories separately as described above, we estimated a ‘covariate-only’ baseline model for each outcome. Then, the best fit models within each category were compared to the baseline model, and to each other. Descriptive fit indices in Table 1 include: 1) the *Akaike* information criterion (*AIC)*, a ‘complexity-penalized’ log-likelihood based measure of ‘unexplained information’ in a model, where smaller values are preferred, 2) the concordance statistic (c-stat), a measure of predictive accuracy of the model, and 3) a pseudo R^2^ as an estimate of total variability explained by the model.

**Table 1:** Descriptive Results for Best-Fit Models Within Each Model Category

	*Depression*			*Cardiac Disease*			*Obesity*		
Model Type	R^2^	AIC	c-stat	R^2^	AIC	c-stat	R^2^	AIC	c-stat
Multiple Individual risk	.197	47,899	.745	.200	27,970	.798	.042	67,013	.609
*Best-fit*	*11 items, ‘once’*			*11 items, ‘more than once’*			*11 items, ‘once’ (by AIC)*		
Cumulative Risk – Categorical	.178	48,638	.734	.200	27,974	.797	.041	67,035	.608
*Best-fit*	*11 items, ‘once’ + quadratic*			*11 items, ‘more than once’*			*11 items, ‘once’ (by AIC)*		
Cumulative Risk –Continuous	.176	48,645	.732	.199	27,979	.796	.041	67,037	.608
*Best-fit*	*9 items, ‘once’ + quadratic*			*11 items, ‘more than once’*			*11 items, ‘once’ (by AIC)*		

R^2^ is Nagelkerke. (by AIC) indicates that the model comparisons within that model category were not significantly different from each other, and the best fit model was chosen as that with the smaller AIC. For the depression outcome only, the CR continuous model included a quadratic term

In addition to inspection of the descriptive fit indices, we performed hypothesis testing for model selection using the two-step approach introduced by Vuong for hypothesis testing of differences in non-nested (or partially non-nested) models [29]. Nested model comparisons using the likelihood ratio test are common, for example in instances of comparing two regression models where the second contains all the predictors from the first, except one. Non-nested models are defined as pairs (or sets) of models where one model cannot be obtained by introducing a restriction or constraint on the other model. Because all models compared here include the same set of covariates, but different characterizations of the ACE predictor, they are classified as partially non-nested. The Vuong approach first tests for model distinguishability via the Ω test (the ratio of the log-likelihoods of the models, obtained from the Kullback-Leibler information criteria). Distinguishability implies a population-based (not just sample-based) difference in fit. If significant, indicating distinguishability, it is followed by Vuong’s closeness test (a z-test of the difference in model predicted probabilities) to test for differences in the fit of distinguishable models [29]. When models were found to be non-significantly different via the Vuong’s formal model comparison tests, model selection was based on comparing the AICs using a rule-of-thumb of differences in AICs > 50 considered substantial support for the model with the smaller AIC [30].

All data and R scripts are available on request from the first author.

## Results

Full results for all models and model comparisons, including the comparisons used to arrive at the best fit model within each category, can be found in the Additional file 1: Appendix, Tables A – C.

Descriptive results for the best fit model within each of the three model categories and for each outcome are shown in Table 1.

### Model comparisons

All model comparisons between the covariate-only baseline model and models including any ACE predictor found significantly better fit for models including any ACE predictor. For the depression outcome, the best fitting model was the MIR model, with a large magnitude of difference between the models in terms of Vuong’s test, as well as reduction in AIC (− 739, a substantial difference [30]) and increased predictive power (21% increase in R^2^ and 17% improvement in the c-statistic).

For the cardiac disease and obesity outcomes, the MIR model was ‘distinguishable’ but of equal fit to the CRCat model, and both were of significantly better fit than the CRCn model. The results of the between-category model comparisons are shown in Table 2.

**Table 2:** Model Comparison Results

Outcome	Model	AIC	Covar	MIR	CRCat
Depression	Covar	51,519	1		
	MIR	47,899	.067***	1	
			−29.58***		
	CRCat	48,638	.052***	.019***	1
			−26.76***	11.34***	
	CRCn	48,645	.051***	.018***	.001***
			−26.66***	12.21***	2.51***
Cardiac	Covar	28,232	1		
Disease	MIR	27,968	.006***	1	
			−7.72***		
	CRCat	27,974	.006***	.001***	1
			−7.61***	**NDF**	
	CRCn	27,979	.005***	.001***	.001**
			−7.49***	2.75**	2.24***
Obesity	Covar	67,186	1		
	MIR	67,013	.004***	1	
			−6.92***		
	CRCa	67,035	.003***	.001***	1
			−6.54***	**NDF**	
	CRCn	67,037	.003***	.001*	.001***
			−6.10***	3.30***	2.39**

*Covar* covariate only model with no ACE predictors, *MIR* multiple individual risk mode, *CRCat* cumulative risk (ACE Score) categorical, *CRCn* cumulative risk (ACE Score) continuous

Within-cell values are the test statistics for Ω (top) and Vuong’s test (bottom). Positive values of Vuong’s indicate the model in the column was better fitting, negative values indicate the model in the row was better fitting

*NDF* Non Different Fit

*p < .05

**p < .01

***p < .001

### Model results & inferences

#### Cumulative ACE score models

For the depression and obesity outcomes, the best fit model included all 11 questions, exposure coded for responses of ‘once’. For the cardiac disease outcome the best fit model also included all 11 questions, exposure coded for ‘more than once’. Estimation of these models for all three outcomes found significance for every level (1–11) of the cumulative ACE Score predictor compared to the zero category (all *p*-values < .001, coefficients not shown).

#### Multiple Individual Risk (MIR) models

Each of the unadjusted relationships between individual ACE predictors and the outcomes were significant (unadjusted for other ACEs but including the covariates).

As can be seen in Table 3, inferences about the relationships between the srACEs and outcomes are different under the MIR model, and patterns of relationships in the adjusted models suggest that different specific srACEs are related to each outcome. For the depression outcome, 8 of the 11 ACEs had a significant relationship, with the question ‘Did you live with anyone who was depressed, mentally ill, or suicidal?’ showing the strongest association (*OR* = 2.89 [2.74, 3.07]). For the obesity outcome, only 4 of the srACEs were significantly related, including 2 of the sexual abuse questions, and the strongest association was with the ‘emotional abuse’ question ‘How often did a parent or adult in your home ever swear at you, insult you, or put you down?’ (*OR* = 1.18 [1.12, 1.24]). The variance inflation factors for the MIR model were all well below acceptable thresholds [31].

**Table 3:** MIR Model Results

	Depression – MIR	Unadjusted association	Cardiac Disease - MIR	Unadjusted association	Obesity - MIR	Unadjusted association
Household Mental Illness	2.89***	4.08***	*.95*	1.26***	*1.01*	1.14***
Household Alcoholism	1.20***	1.97***	*1.05*	1.30***	*1.02*	1.12***
Household Drug Abuse	*.96*	2.14***	1.30***	1.73***	*.76*	1.14***
Household Criminal	*1.04*	1.95***	1.36***	1.82***	*1.08*	1.21***
Divorce	.93*	1.41***	*1.05*	1.23***	*.99*	1.07***
Household Violence	*.98*	2.01***	1.13*	1.52***	*1.02*	1.19***
Physical Abuse	1.29***	2.51***	1.18**	1.59***	1.09*	1.27***
Emotional Abuse	1.46***	2.37***	1.19***	1.46***	1.11***	1.21***
Sexually Touched	1.74***	3.11***	*.87*	1.56***	1.18**	1.43***
Sexual Touching	1.13*	3.02***	1.46***	1.93***	*1.04*	1.49***
Forced Sex	1.28***	3.60***	1.27*	2.07***	1.08*	1.53***
VIFS^1^	1.16–2.13		1.07–2.11		1.09–1.45	

Italicized coefficients are non-significant

^1^VIFS = range of the variance inflation factors obtained for the srACE predictors in the covariate adjusted MIR model

*p < .05

**p < .01

***p < .001

## Discussion

Our primary goal in this research was to evaluate the fit and performance of a ‘multiple individual risk’ model, where all ACE events are separately entered into a single prediction model, in contrast to a ‘cumulative risk model’ approach for predicting adult health outcomes. This research was motivated by observation that the cumulative risk model, while a statistically powerful and parsimonious approach [8], may not be necessarily the best characterization of the impacts of childhood adversity on adult health for all outcomes because it obscures the relative contributions of individual adversity event types.

In contrast, a multiple risk model, while sacrificing information about the general impact of an accumulation of events, will yield information about the relative strength of the associations between individual event types and outcomes. The multiple individual risk model is also more sensitive in that it can allow frequency and severity of specific events to be considered in a statistical model when such information is available, while in a cumulative risk approach a threshold has to be defined for ‘exposure’. Timing, frequency and severity of adverse events are known risk factors for several adult outcomes [32].

Despite the additional information gained from application of a multiple individual risk model, it is virtually absent from the literature, despite the long history of research into the effects of specific abuse types (‘single adversity approaches’ [6]). For example, there is substantial theoretical and empirical support for childhood sexual abuse specifically (compared to other childhood adversities) as most strongly predictive of several outcomes including suicidality [32], cardiopulmonary symptoms, and obesity [33]. The same is supported for the importance of childhood neglect in predicting cognitive outcomes, contrasted with physical abuse specifically because of differences in the amount of stimulation the abused child receives [34]. Importantly though, this earlier body of research most often did not model the co-occurrence of other individual risk events, even though as early as Rutter’s seminal work it was recognized that an adverse childhood environment tends to include many interacting sets of events and circumstances [35]. This finding has been consistent from within the ACEs framework literature [20, 36] and preceding it [35]. The multiple individual risk model allows for a more nuanced assessment of the effects of specific adversities when those adversities do not occur in isolation.

We found that the multiple individual risks model was a significantly better fit to the data for the lifetime history of depression outcome only. In addition to the significant difference in fit found via hypothesis testing, the MIR model accounted for 21% more variability in the outcome by R^2^, and an increase in model predictive performance of 17% by the c-statistic. In the case of the other two outcomes, the multiple individual risks model and the cumulative risk model (with categorical coding) were population distinguishable, but not of different fit, and inspection of the other model fit indices reveal little difference in their performance.

This is an intriguing finding that may reflect the fact that among the outcomes we analyzed, current depression may be most strongly related to biased recall for childhood events [37]. Also, obesity and cardiac disease can be construed as more ‘biological’ outcomes than depression, and it may be the case that it is, in fact, an accumulation of adversity that predicts ill physical health, but that specific individual events are more strongly predictive of mental health outcomes. This possibility goes unexamined when the cumulative ACE Score is analyzed without a multiple individual risk model analyzed as well.

In the course of the model comparisons in this study, we arrived at a statistically best fit model within each category. For comparisons between models with 11 items (with the 3 sexual ACEs counted separately) and models with 9 items, we found that in all but one case an 11 items model fit better. The exception was in the case of the continuous variable treatment for the depression outcome, which we suspect may be an artifact of the need to include a quadratic term in that model. We also found that coding individuals as exposed who responded that the reported events happened ‘more than once’ was the best fit for the cardiac disease outcome only, for the other two outcomes the response of ‘ever’ happened was the best fit.

For all three outcomes the continuous score treatment (in the cumulative risk model) performed worst. Given the additional statistical and theoretical assumptions required to employ a continuous cumulative risk model, it seems an untenable approach. Overall, we conclude that utilizing the available ACE event predictors with as much information as possible by using all 11 is a reasonable approach in large-sample data sources.

Taken together, we interpret these results as suggesting that investigators working with large srACEs data sources should empirically derive the number of items, as well as the exposure coding strategy, that are a best fit for the outcome under study. These analytic processes should be reported in order to improve the rigor and reproducibility of findings. From the perspective of information gained, these analytic choices can be seen not just as initial steps in data analysis, but also that their result confers additional information about the relationship between adversity and outcomes. Additionally, we suggest that unadjusted univariate associations between ACEs and outcomes (which are often reported in research publications) be supplemented with or replaced by estimation of the ‘multiple individual risk’ model in studies that implement a cumulative ACE Score. This process yields additional information about ACE-health relationships.

### Limitations

The primary purpose of this study was to compare predictor characterizations, not to draw conclusions about the effects of ACEs. We therefore only included cases with complete data on all the predictors and the outcomes in order to avoid inconsistent listwise deletions across models, resulting in a loss of 17.9% of the data. Because of this decision we were unable to estimate the models using the survey design weighting appropriate for drawing population-true point estimates or relational inferences. Therefore, our model results in terms of the point estimates of ACE predictors should be interpreted with this caveat. Similarly, we used the same set of covariates in every model, even when they may not have been significantly related to the outcome or may have been collinear with each other or with the ACEs predictors. Model fit may have been influenced by this decision, but we know that the differences between models was attributable to differences in ACE predictor characterizations, not to variations in covariates or the unequal distribution of survey weights.

## Conclusions

In this work, we have highlighted only two possible models for the effects of adversity on outcomes (CR vs MIR) but there are numerous others. Some research frames the srACEs in a psychometric context, treating them as indicators of underlying latent variables and applying techniques like exploratory and confirmatory factor analysis [38, 39]. Some researchers working in the ACEs Framework have begun conceptualizing how ACEs might be related to outcomes by applying models like cluster analysis [40], latent class [41] or recursive partitioning [42] to classify people into groups, rather than classify ACEs into scores. Results obtained from these differing conceptualizations also differ in what they imply about how adversity and trauma impact individuals, and researchers are advised to include these modeling considerations in their discussions. Model fit approaches such as we utilized here can guide researchers in choosing an operationalization specific to the data.

## Supplementary information

**Additional file 1: Table A.** Model Comparison Results for Obesity Outcome. **Table B.** Model Comparison Results for Cardiac Disease Outcome. **Table C.** Model Comparison Results for Lifetime Depression Outcome.

## Data Availability

The data supporting the results in this study are publically available through the CDC’s website at https://www.cdc.gov/brfss/index.html. The specific analytic files and R scripts used to analyze the data are available upon request from the first author.

## Abbreviations

ACEs: Adverse Childhood Experiences
AIC: *Akaike* information criterion
BMI: Body Mass Index
BRFSS: Behavioral Risk Factor Surveillance System
CDC: Centers for Disease Control and Prevention
CR: Cumulative Risk
CrCat: Cumulative Risk, Categorical
CrCn: Cumulative Risk, Continuous
c-stat: Concordance Statistic
LCA: Latent Class Analysis
MIR: Multiple Individual Risk
VIF: Variance inflation factor
