# Supplementary material for: Contrasting cumulative risk and multiple individual risk models of the relationship between Adverse Childhood Experiences (ACEs) and adult health outcomes
Source: BMC Med Res Methodol. 2020 Sep 29;20:239. doi: 10.1186/s12874-020-01120-w (PMC7525970; doi:10.1186/s12874-020-01120-w)
Supplement: Supplementary file 1 — Additional file 1: Table A. Model Comparison Results for Obesity Outcome. Table B. Model Comparison Results for Cardiac Disease Outcome. Table C. Model Comparison Results for Lifetime Depression Outcome. [file 12874_2020_1120_MOESM1_ESM.docx]

Table A – Model Comparison Results for Obesity Outcome

|  | | | *Model Category* | | | | | | | | | | | | | |
| --- | --- | --- | --- | --- | --- | --- | --- | --- | --- | --- | --- | --- | --- | --- | --- | --- |
|  |  |  |  | MIR | | | | CrCat | | | | | CrCn | | | |
|  | AIC  R^2^ | *Model* | Covar | **±**  11, 1x | 11, >1x | 9, 1x’ | 9, >1x | **±±**  11, 1x | 11, >1x | 9, 1x | 9, >1x | **±**  11, 1x | | 11, >1x | 9, 1x |  |
|  | 67186  .038 | covar | 1 |  |  |  |  |  |  |  |  |  | |  |  |  |
|  |  |  |  |  |  |  |  |  |  |  |  |  | |  |  |  |
| MIR | 67013  .042 | 11, 1x | .004*** | 1 |  |  |  |  |  |  |  |  | |  |  |  |
|  |  |  | -6.92*** |  |  |  |  |  |  |  |  |  | |  |  |  |
|  | 67023  .042 | 11, >1x |  | .001*** |  |  |  |  |  |  |  |  | |  |  |  |
|  |  |  |  | NDF |  |  |  |  |  |  |  |  | |  |  |  |
|  | 67024  .042 | 9, 1x |  | .001*** |  | 1 |  |  |  |  |  |  | |  |  |  |
|  |  |  |  | 2.57** |  |  |  |  |  |  |  |  | |  |  |  |
|  | 67024  .042 | 9, >1x |  |  |  | .001* |  |  |  |  |  |  | |  |  |  |
|  |  |  |  |  |  | NDF |  |  |  |  |  |  | |  |  |  |
| CrCat | 67035  .041 | 11, 1x | .003*** | .001*** |  |  |  | 1 |  |  |  |  | |  |  |  |
|  |  |  | -6.54*** | NDF |  |  |  |  |  |  |  |  | |  |  |  |
|  | 67052  .041 | 11, >1x |  |  |  |  |  | .001*** |  |  |  |  | |  |  |  |
|  |  |  |  |  |  |  |  | NDF |  |  |  |  | |  |  |  |
|  | 67049  .041 | 9, 1x |  |  |  |  |  | NS |  | 1 |  |  | |  |  |  |
|  |  |  |  |  |  |  |  | NT |  |  |  |  | |  |  |  |
|  | 67063  .041 | 9, >1x |  |  |  |  |  |  |  | .001*** | 1 |  | |  |  |  |
|  |  |  |  |  |  |  |  |  |  | NDF |  |  | |  |  |  |
| CrCn | 67037  .041 | 11, 1x, | .003*** | .01* |  |  |  | .001*** |  |  |  | 1 | |  |  |  |
|  |  |  | -6.10*** | 3.30*** |  |  |  | 2.39** |  |  |  |  | |  |  |  |
|  | 67047  .041 | 11, >1x |  |  |  |  |  |  |  |  |  | .001*** | | 1 |  |  |
|  |  |  |  |  |  |  |  |  |  |  |  | NDF | |  |  |  |
|  | 67060  .041 | 9, 1x |  |  |  |  |  |  |  |  |  | .001*** | |  | 1 |  |
|  |  |  |  |  |  |  |  |  |  |  |  | 4.95*** | |  |  |  |
|  | 67068  .041 | 9, >1x |  |  |  |  |  |  |  |  |  |  | |  |  |  |
|  |  |  |  |  |  |  |  |  |  |  |  |  | |  |  |  |

Table B – Model Comparison Results for Cardiac Disease Outcome

|  |  | | *Model Category* | | | | | | | | | | | | | |
| --- | --- | --- | --- | --- | --- | --- | --- | --- | --- | --- | --- | --- | --- | --- | --- | --- |
|  |  |  |  | MIR | | | | CrCat | | | | CrCn | | | | |
|  | AIC  R^2^ |  | Covar | 11, 1x | ±±  11, >1x | 9, 1x’ | 9, 1x | 11, 1x | ±  11, >1x | 9, 1x | 9, >1x | | 11, 1x | ±  11, >1x | 9, 1x |  |
|  | 28232  .189 | covar | 1 |  |  |  |  |  |  |  |  | |  |  |  |  |
|  |  |  |  |  |  |  |  |  |  |  |  | |  |  |  |  |
| MIR | 27990  .199 | 11, 1x |  | 1 |  |  |  |  |  |  |  | |  |  |  |  |
|  |  |  |  |  |  |  |  |  |  |  |  | |  |  |  |  |
|  | 27968  .201 | 11, >1x | .006*** | .001*** | 1 |  |  |  |  |  |  | |  |  |  |  |
|  |  |  | -7.72 | NDF |  |  |  |  |  |  |  | |  |  |  |  |
|  | 28021  .198 | 9, 1x |  |  |  | 1 |  |  |  |  |  | |  |  |  |  |
|  |  |  |  |  |  |  |  |  |  |  |  | |  |  |  |  |
|  | 27994  .199 | 9, >1x |  |  | .001*** | .001*** |  |  |  |  |  | |  |  |  |  |
|  |  |  |  |  | 3.02** | -2.29** |  |  |  |  |  | |  |  |  |  |
| CrCat | 27994  .199 | 11, 1x |  |  |  |  |  | 1 |  |  |  | |  |  |  |  |
|  |  |  |  |  |  |  |  |  |  |  |  | |  |  |  |  |
|  | 27974  .199 | 11, >1x | .006*** |  | .001*** |  |  | .001*** | 1 |  |  | |  |  |  |  |
|  |  |  | -7.61*** |  | NDF |  |  | -1.67* |  |  |  | |  |  |  |  |
|  | 28032  .198 | 9, 1x |  |  |  |  |  |  | .001** | 1 |  | |  |  |  |  |
|  |  |  |  |  |  |  |  |  | 4.82*** |  |  | |  |  |  |  |
|  | 28016  .199 | 9, >1x |  |  |  |  |  |  | .001** | .001*** |  | |  |  |  |  |
|  |  |  |  |  |  |  |  |  | 4.84*** | -1.69* |  | |  |  |  |  |
| CrCn | 27992  .197 | 11, 1x |  |  |  |  |  |  |  |  |  | | 1 |  |  |  |
|  | 27979  .198 | 11, >1x | .005*** |  | .001*** |  |  |  | **.001**** |  |  | | .001*** | 1 |  |  |
|  |  |  | -7.49*** |  | 2.75** |  |  |  | **2.24***** |  |  | | -1.42 |  |  |  |
|  | 28026  .197 | 9, 1x |  |  |  |  |  |  |  |  |  | |  |  | 1 |  |
|  | 28013  .198 | 9, >1x |  |  |  |  |  |  |  |  |  | |  | .001*** | .001*** |  |
|  |  |  |  |  |  |  |  |  |  |  |  | |  | 5.07*** | -1.65* |  |

Table C– Model Comparison Results for Lifetime Depression Outcome

|  |  | | *Model Category* | | | | | | | | | | | | |  |
| --- | --- | --- | --- | --- | --- | --- | --- | --- | --- | --- | --- | --- | --- | --- | --- | --- |
|  |  |  |  | MIR | | | | CrCat | | | | | CrCn (+) | | |  |
|  | AIC  R^2^ | *Model* | covar | ±±  11, 1x | 11, >1x | 9, 1x | 9, >1x | ±  11,1x | 11, >1x | 9, 1x | 9, >1x | ±  11, 1x | | 11, >1x | 9, 1x | |
|  | 51519  .104 | covar | 1 |  |  |  |  |  |  |  |  |  | |  |  | |
|  |  |  |  |  |  |  |  |  |  |  |  |  | |  |  | |
| MIR | 47899  .197 | 11, 1x | .067*** | 1 |  |  |  |  |  |  |  |  | |  |  | |
|  |  |  | -29.58*** |  |  |  |  |  |  |  |  |  | |  |  | |
|  | 48033  .194 | 11, >1x |  | .005*** | 1 |  |  |  |  |  |  |  | |  |  | |
|  |  |  |  | 4.18*** |  |  |  |  |  |  |  |  | |  |  | |
|  | 47878  .198 | 9, 1x |  | .01** |  | 1 |  |  |  |  |  |  | |  |  | |
|  |  |  |  | NDF |  |  |  |  |  |  |  |  | |  |  | |
|  | 48013  .194 | 9, >1x |  |  |  | *.005**** | 1 |  |  |  |  |  | |  |  | |
|  |  |  |  |  |  | *3.87**** |  |  |  |  |  |  | |  |  | |
| CrCat | 48638  .179 | 11, 1x | .052*** | .019*** |  |  |  | 1 |  |  |  |  | |  |  | |
|  |  |  | -26.76*** | 11.34*** |  |  |  |  |  |  |  |  | |  |  | |
|  | 48783  .175 | 11, >1x |  |  |  |  |  | .005*** | 1 |  |  |  | |  |  | |
|  |  |  |  |  |  |  |  | 4.42*** |  |  |  |  | |  |  | |
|  | 48740  176 | 9, 1x |  |  |  |  |  | .001*** |  | 1 |  |  | |  |  | |
|  |  |  |  |  |  |  |  | 6.12*** |  |  |  |  | |  |  | |
|  | 48846  .173 | 9, >1x |  |  |  |  |  |  |  | .004*** | 1 |  | |  |  | |
|  |  |  |  |  |  |  |  |  |  | 3.94*** |  |  | |  |  | |
| CrCn  (+) | 48645  .178 | 11, 1x | .051*** | .019*** |  |  |  | .001*** |  |  |  | 1 | |  |  | |
|  |  |  | -26.66*** | 11.34*** |  |  |  | 2.51*** |  |  |  |  | |  |  | |
|  | 48796.174 | 11, >1x |  |  |  |  |  |  |  |  |  | .004*** | | 1 |  | |
|  |  |  |  |  |  |  |  |  |  |  |  | 4.75*** | |  |  | |
|  | 48748  .176 | 9, 1x |  |  |  |  |  |  |  |  |  | .001*** | |  | 1 | |
|  |  |  |  |  |  |  |  |  |  |  |  | 6.19*** | |  |  |  |
|  | 48858  .173 | 9, >1x |  |  |  |  |  |  |  |  |  |  | |  | .004*** | |
|  |  |  |  |  |  |  |  |  |  |  |  |  | |  | 3.76*** | |

(+) all continuous score models for this outcome were fit with a quadratic term

Notes for Tables A-C.

MIR = ‘multiple individual risk’ model, CRCat = ‘cumulative risk (ACE Score), categorical, CrCn = = ‘cumulative risk (ACE Score), continuous

Descriptive statistics for all models shown in the far-left column.

‘9’, ‘11’ refer to the number of srACEs questions included in the model. ‘1x’ means exposure was coded for responses that the event happened’ once’, ‘>1x’ means exposure was coded for responses that the event happened ’’more than once’, for those questions with frequency response options.

Covar = covariate only model with no ACE predictor.

Within-cell values are the test statistics for Ω (top) and Vuong’s test (bottom). Positive values of Vuong’s indicate the model in the column was better fitting, negative values indicate the model in the row was better fitting.

**±** indicates best fitting model in that model category, **±±** indicates best fitting model overall

NS = Non-Significant, NDF = Non-Different Fit, NT = Not Tested, *p<.05, **p<.01, ***p<.001, ****p<.0001
